# Automated Detection of Brain Tumor through Magnetic Resonance Images Using Convolutional Neural Network

**DOI:** 10.1155/2021/3365043

**Published:** 2021-11-30

**Authors:** Sahar Gull, Shahzad Akbar, Habib Ullah Khan

**Affiliations:** ^1^Riphah College of Computing, Riphah International University, Faisalabad Campus, Faisalabad 38000, Pakistan; ^2^Department of Accounting and Information Systems, College of Business and Economics, Qatar University, Doha, Qatar

## Abstract

Brain tumor is a fatal disease, caused by the growth of abnormal cells in the brain tissues. Therefore, early and accurate detection of this disease can save patient's life. This paper proposes a novel framework for the detection of brain tumor using magnetic resonance (MR) images. The framework is based on the fully convolutional neural network (FCNN) and transfer learning techniques. The proposed framework has five stages which are preprocessing, skull stripping, CNN-based tumor segmentation, postprocessing, and transfer learning-based brain tumor binary classification. In preprocessing, the MR images are filtered to eliminate the noise and are improve the contrast. For segmentation of brain tumor images, the proposed CNN architecture is used, and for postprocessing, the global threshold technique is utilized to eliminate small nontumor regions that enhanced segmentation results. In classification, GoogleNet model is employed on three publicly available datasets. The experimental results depict that the proposed method is achieved average accuracies of 96.50%, 97.50%, and 98% for segmentation and 96.49%, 97.31%, and 98.79% for classification of brain tumor on BRATS2018, BRATS2019, and BRATS2020 datasets, respectively. The outcomes demonstrate that the proposed framework is effective and efficient that attained high performance on BRATS2020 dataset than the other two datasets. According to the experimentation results, the proposed framework outperforms other recent studies in the literature. In addition, this research will uphold doctors and clinicians for automatic diagnosis of brain tumor disease.

## 1. Introduction

**Figure 1:**
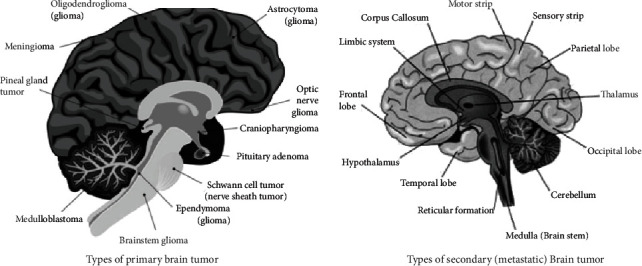
Categories of brain tumor.

Brain tumor is also called intracranial cancer in which growth of abnormal cells in the brain tissues happened [1]. According to the National Brain Tumor Society (NBTS), more than 4200 patients in the UK suffer from primary brain tumors, and in the USA, 1300 patients died each year due to brain tumor [2]. In 2015, approximately 29000 patients endured primary brain tumors in the USA [3]. An estimated 17,760 deaths occurred, and 23,820 new brain tumor cases were predicted in the United States (US) in 2019 [4]. According to 2021 cancer statistics, 24,530 cases of brain tumor predicted till now include 13,840 men and 10,690 women in the US [5]. Brain tumors fall into two categories: primary brain tumors and secondary (metastatic) brain tumors [6]. A primary brain tumor is defined as one that has not spread to other parts of the body. Primary brain tumors can be malignant or benign [7]. The gradual development of benign brain tumors has multiple limitations and slowly grows. Benign tumors could be life-threatening, and malignant brain tumors develop fast, have abnormal regions, and prevail in other brain areas, even though the malignant cells are not at a critical region. Metastatic brain tumors start anywhere in the body as the tumor and brain prevalence [8]. The World Health Organization (WHO) built a ranking system to standardize connectivity and estimate brain tumor outcomes. Brain tumors have more than 120 types: meningioma, epidermoid, medulloblastoma, lymphoma, pituitary adenoma, glioma, oligodendro-glioma, and glioblastoma multiforme are certain common types of brain tumor [9]. According to researchers, brain tumor is about 80% made up of gliomas [10]. Glioma is generally categorized as benign or malignant glioma. The WHO subsequently classified gliomas into four classes between grade I and grade IV [11]. Grades I and II, also called low-grade gliomas (LGG), have a longer lifespan than grades III and IV, which are high-grade gliomas (HGG). Low-grade gliomas can develop over time into higher-grade gliomas [12]. The glioma 45%, meningioma 15%, and pituitary tumor 15% exist among all brain tumors. According to 2018 global cancer statistics, there were 296,851 new cases of brain cancer and 241,037 deaths due to compromised nervous system in Asia [13]. Recently, according to global cancer statistics of 2020, there were 308,102 new cases of brain cancer and 251,329 deaths in Asia [14], and these numbers are increasing every day. The types of brain tumor have been shown in Figure 1 [6].

**Figure 2:**
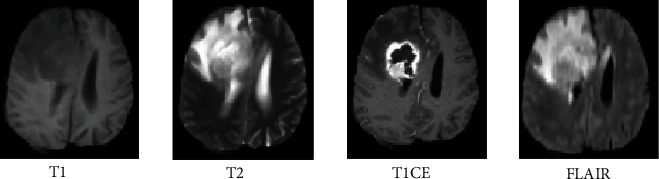
Four multisequence MR images.

Common treatment methods largely rely on medical techniques, such as magnetic resonance imaging (MRI), computed tomography (CT), and X-Rays [15]. MRI is a severe strategy of medical imaging used to treat brain tumors with high-resolution images [16]. Different modalities are used for brain tumor detection, while MR images provide the most meaningful information on a brain tumor. The common multimodels of MRI brain tumors are T1, T2, T1CE, and FLAIR [17]. The T1 images recognize tissues; however, T2 MR images treat the edema region with positive signals in the image. In T1CE images, tumor edges are found without the incredible signal of the experts in the complex tumor tissue cells (gadolinium particles) [18]. The remarkable hyposection of the tumor mass suggests that it can only be separated by a similar cell area technique since the necrotic cells cannot be distinguished from the surrounding areas [19]. In FLAIR images, molecular water signals are silenced, which allows the CSF area to be detected [20]. Four multisequence MR images [21] (T1, T2, T1CE, and FLAIR) are shown in Figure 2.

Artificial intelligence in brain tumor [22] is being utilized in many areas of research by radiologists. The diagnosis of brain tumor is performed in a variety of ways through transfer learning [23, 24] and deep learning methodologies [25–28], such as CNN architecture is employed for the segmentation and classification [29–31]. In radiology, these methods provide a great deal of awareness of diagnosis, treatment, and perception [32]. This study is aimed at providing the solution to existing problems of segmentation and generating a high-quality outcome with less computation time and error rate, using transfer learning-based classification without the use of specialized hardware, which is not accessible in underdeveloped countries with multiple image processing tasks equipped for MRIs with focal disabilities [33]. Therefore, the developed method is efficient and reliable for the automated detection of brain tumor. In the proposed method, the entire CNN model GoogleNet is adopted for classification.

It has become evident that the DL approaches for the detection of brain tumor are more effective than conventional methods [34]. The CNN-based DL model shows promising results in the diagnosis of tumors through MR images [35]. In the previously automated approaches [36–38], the authors used the preprocessing stage to boost the region of interest (ROI), which enhances the detection accuracy of traditional methods such as GrabCut and watershed. Without preprocessing procedures, traditional techniques do not perform well.

The segmentation and classification were performed by DL in the related work, and several pretrained CNN models were used for the brain tumor detection. In the literature, the pretrained models (VGG-16, AFPNet, Dense U-Net, ResNet 50, and AlexNet) were used. The segmentation of tumors is a challenging problem as tumors vary greatly in size, shape, and intensity. The limitation of previous studies are the inadequate anticipating of this segmentation problem. Previous approaches rely on manually segmented tumor regions which are invasive and time-consuming. Traditional algorithms and their variations were not able to considerably improve the performance. Moreover, the existing techniques had been tested and trained on small and local datasets with inadequate representation of all tumor classes. Accurate classification is a difficult research problem that can be effectively addressed with a CNN model [39]. The benefit of CNN classifiers is that they did not require manual classification and provided a completely automatic classification. There is a pressing need to develop fully automated brain tumor detection using MR images which require robust classification of brain tumors. Therefore, a fully automated DL model is proposed that segments the tumor and further classifies it. In this paper, we address the issues of incorrect segmentation and ineffective classification using CNN-based models. In classification, the transfer learning techniques are applied on CNN model using GoogleNet for brain tumor classification. For radiology research and experiments, we performed transfer learning, fine-tuning, and freezing techniques to reduce the parameters. The fully connected layer has been replaced rendering to the dataset label. In addition, to transfer learning, high processing power from GPU is required to train smoothly which is cost-effective. An additional drawback in transfer learning is that the image input size is fixed. In this work, we have adjusted MR images according to the pretrained model input size.

The major contributions of this paper are as follows:

- A new deep learning model is proposed for brain tumor segmentation and classification
- In segmentation, the proposed model consists of different CNN-based layers which are trained on the latest BRATS2020 dataset. The preprocessing is performed using a median filter, and postprocessing is performed using global threshold technique for achieving better results
- A large number of training samples are utilized to improve the performance of proposed method
- In proposed model, the focal loss function is used to resolve class unbalance issues, and batch normalization is performed to avoid overfitting problems
- In the proposed framework, transfer learning techniques are applied on pretrained GoogleNet model for brain tumor classification
- The proposed method is computationally effective and achieved more accurate and reliable results which are better than state-of-the-art methods for segmentation and classification of brain tumor

The remaining paper is organized as follows: Section 2 elaborates the related work that investigates the existing models, techniques, and working of previous methods. In Section 3, the comprehensive details of the developed framework are described for brain tumor detection. The proposed solution based on CN architecture includes the preprocessing, segmentation, postprocessing, and classification and is supported to resolve existing issues of brain tumors.  Section 4 defines the measurement metrics, findings, and experiment results of the proposed methodology.  Section 5 provides a discussion and critical analysis of the proposed methodology.  Section 6 summarizes the findings and provides future directions of this domain.

## 2. Literature Review

Currently, DL strategies are being used to help identify tumor segments and successive mapping of brain tumor shape and texture and estimate the survival rate of patients based on MR image datasets [40]. Different CNN architectures have been developed to segment and classify brain tumor.

Hu et al. [41] presented a method based on the MCCNN to take out more distinctive multiscale features for the brain tumor segmentation and linked conditional random fields (CRFs). Three models were developed and performed with different perspectives using 2D patches to obtain an overall segmentation outcome. The proposed approach was tested on all three public databases. The outcomes showed that complete tumor (CT) was 0.88, tumor core (TC) 0.81, enhanced tumor (ET) 0.76 for DSC, the CT 0.86, TC 0.81, and ET 0.69 for PPV, and CT 0.90, TC 0.84, and ET 0.86 for sensitivity on the dataset BRATS2013. The outcome was CT 0.87, TC 0.76, and ET 0.75 for DSC; 0.88, 0.83, and 0.75 for PPV; and 0.87, 0.74, and 0.80 for sensitivity on the BRATS 2015, and the result of the purposed method showed the ET 0.7178, WT 0.8824, and TC 0.7481 for DSC; the ET 0.8684, WT 0.9074, and TC 0.7621 for sensitivity; the WT 0.9918, ET 0.9947, and TC 0.9969 for specificity; and the ET 5.6864, TC 9.6223, and WT 12.6069 for HD on BRATS 2018 dataset.

Zhou et al. [42] developed a model based on the CNN method utilized for segmentation. This study provides the solution to two main issues, the first problem was the lack of spatial information, and the second problem was insufficient multiscale process capability. The 3D Atrous was used to minimize the first query. In the background framework, pyramid, to integrate the backbone to solve the second issue of 3D Atrous, the results of the proposed model show WT 0.83, TC 0.68, and ET 0.60 on the BRATS2013 dataset; the WT 0.82, TC 0.68, and ET 0.60 on the BRATS2015 dataset; and WT 0.8658, TC 0.7688, and ET 0.74434 on the BRATS2018 dataset.

Agerwal et al. [43] developed a model for classification based on transfer learning. A DL model was built that categorizes the MR images into brain tumor affected and standard images. In this study, the proposed CNN architecture used the VGG16 model that classified the MR images into two classes. The outcomes showed that the proposed model attained 96.5% accuracy in training and 90% accuracy in testing with low complexity on the publicly available dataset.

Laukamp et al. [44] developed a multiparametric DL model to examine the performance of automated detection of meningiomas. The MR image dataset was used to detect the meningiomas. The deep learning model (DLM) was used on an independent dataset and the BRATS benchmark dataset for the brain tumor in glioma cases. The findings of this suggested technique showed that, for T1CE, the range was 0.46-0.93, total tumor volume 0.81 ± 0.10, contrast-enhancing volume 0.78 ± 0.19, and range 0.27-0.95 on the BRATS dataset.

Indra and Yessi [45] presented a model based on the GLCM approach used for feature extraction and the *T*-test approach for classification. The light signal from the brain was transformed into a grey matrix before eliminating the features. The experiment was based on 40 test results. The GLCM technique created an image of the brain and abnormal brain by extracting features. It was found that each character has a *P* value < 0.05, which indicated that the extracted features were used for brain tumor classification on the public dataset.

Akil et al. [46] presented a model based on the CNN model for automatic segmentation of glioblastoma brain tumor. A selective attention technique enhanced the extracted features of MR images. The spatial imbalance relationship was used as an equal sample of image patches to solve the class imbalance problem. The radiologist's dice score range was 74 to 85%. The outcomes showed that the median dice score of the WT was 0.90, TC 0.83, and ET 0.83, respectively, on the BRATS2018 dataset.

Bangalore et al. [47] proposed a model based on DL for the segmentation of brain tumor. The developed method used to simplify the complicated problems of multistage segmentation was the designed 3D-Dense U-Net to break up the binary segmentation problem. The outcomes showed that with the proposed method, the dice score was 0.80, WT was 0.92, and CT was 0.84 on the BRATS2015 dataset, respectively. Similarly, the dice score was 0.90, WT was 0.80, and CT was 0.78 on the BRATS2017 dataset, respectively. For the BRATS2018, the dice score was 0.90, WT was 0.82, and CT was 0.80 for brain tumor segmentation.

Thaha et al. [48] developed a BAT algorithm based on a CNN-based model for segmentation. In the CNN-based model, the small kernels assigned less weight to the framework, positively affecting excess. Preprocessing was done using skull stripping and improved image quality and removed noise. The analysis indicates that the proposed model was a better performance than the existing techniques. The outcomes depicted that the accuracy of E-CNN was 92%, precision 87%, and recall 90% for segmentation on the BRATS2015 dataset.

Talo et al. [49] developed a method to classify the MR images with the VGG-16, Alex-Net, ResNet-34, ResNet-18, and ResNet-50. Pretrained deep learning models are used in tissues with normal, neoplastic, cerebrovascular, degenerative, and inflammatory appearance. This method was automated, which was used to extract and classify features. Data was collected from the Harvard Medical School dataset for 1074 MR images. The data from the Harvard Medical School 1074 MR images were used. The suggested solution was tested and achieved the best results on large MR images of brain tumor. The proposed approach results showed that the accuracy was 95.33% ± 0.6 by the ResNet-50 model.

Sharif et al. [50] employed a method built on the CNN architecture for brain tumor detection. This developed method was performed in two main steps. Firstly, the SbDL model was used for brain tumor segmentation, and another DRLBP fusion technique was used to enhance the functionality through the *particle swarm optimization* (PSO) algorithm. In this study, the Softmax classifier was used for classification purpose. The step of contrast improvement helps to coordinate the division of images, and DRLBP was designed to integrate the functionality for classification. The outcomes showed that the dice score for CT was 88.34%, WT, 91.2%, ET 81.84% on the BRAST2018 dataset, and the average accuracy was more excellent than the 92% using the BRATS2013-BRATS2018 dataset.

Hu et al. [41] developed a method based on the MCCNN to take out more distinctive multiscale features for the brain tumor segmentation and linked CRFs. Three models were developed and performed with different perspectives using 2D patches to obtain an overall segmentation outcome. The proposed approach was tested on all three public databases. The outcomes showed that complete tumor (CT) was 0.88, tumor core (TC) 0.81, and enhanced tumor (ET) 0.76 for DSC; the CT 0.86, TC 0.81, and ET 0.69 for PPV; and CT 0.90, TC 0.84, and ET 0.86 for sensitivity on the dataset BRATS2013. The outcome was CT 0.87, TC 0.76, and ET 0.75 for DSC; 0.88, 0.83, and 0.75 for PPV; and 0.87, 0.74, and 0.80 for sensitivity on the BRATS 2015, and the result of the purposed method showed the ET 0.7178, WT 0.8824, and TC 0.7481 for DSC; WT 0.9918, ET 0.9947, and TC 0.9969 for specificity; ET 0.8684, TC 0.7621, and WT 0.9074 for sensitivity; and the ET 5.6864, TC 9.6223, and WT 12.6069 for HD on BRATS 2018 dataset.

Naser and Jamal [51] developed a DL-based U-Net approach based on the CNN model to detect brain tumor. The VGG-16 model was employed for classification. The Cancer Imaging Archive (TCIA) dataset of 110 LGG MR images was used in this work. The proposed methodology results showed that the DSE was 0.84, and the accuracy of brain tumor detection was 0.92. Additionally, the grading models attained an accuracy of 0.89, sensitivity 0.87, specificity 0.92 at the level of MRI image, and accuracy of 0.95 when compared to the publicly available dataset.

Rundo et al. [52] presented a GTVCUT approach based on cellular automata and adaptive seed selection strategy for brain tumor segmentation. In preprocessing, the contrast stretching operation was performed. In this work, a real dataset was used that included 100 MR images of 25 patients who were affected with a metastatic brain tumor. For the evaluation of the developed GTVCUT approach, different parameters were used. The proposed method achieved 90.88 ± 4.19 DSC, 91.20 ± 7.00 sensitivity, 99.99 ± 0.01 specificity, 0.007 ± 0.008 FPR, and 6.353 ± 6.482 FNR.

Huang et al. [53] developed a differential feature map (DFM) block to detect brain tumor. The squeeze-and-excitation (SE) blocks were concatenated with DFM blocks in the form of a differential feature neural network (DFNN). The proposed approach DFNN classified the brain tumor into two classes (normal and abnormal). The developed framework was trained and tested on two different datasets. The first dataset consisted of more than 10,000 MR images known as database I, and the second dataset consisted of TCGA-LGG dataset known as database II. The outcomes demonstrated that 99% and 97.5% accuracies were attained on database I and database II, respectively, for the proposed DFN approach. On the other hand, for the proposed DFNN approach, 99.2% and 98% accuracies were achieved on database I and database II.

Khalil et al. [54] developed a method called dragonfly algorithm (DA) for segmentation to overcome the problem of variation in tumor structure and size. The preprocessing step is applied on 3D-MR images to extract the tumor edges. Lastly, the two-step DA clustering approach extracted the tumor from all volume MR images through level set segmentation. The publicly available BRATS2017 dataset was used to train and test the proposed approach. The results demonstrated that the proposed method achieved 98.20% accuracy, 95.13% recall and 93.21% precision.

## 3. Proposed Methodology

### 3.1. Analysis of Proposed Framework for Brain Tumor Segmentation

In this section, a fully automated methodology is proposed for segmentation and classification. The developed framework consists of the following steps, preprocessing, skull stripping, segmentation, postprocessing, and classification.

#### 3.1.1. Preprocessing

**Figure 3:**
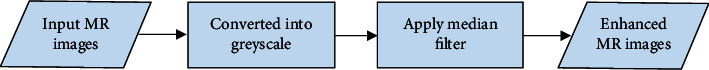
Preprocessing steps of MR images.

The purpose of preprocessing step is to improve image quality and data cleaning and enhance the contrast of MR images. The median filter is used to eliminate the noise and to fetch helpful information. Median filtering is a nonlinear filtering technique employed to retain sharp features during noise filtering in MR images. The preprocessing steps for each MR image are illustrated in Figure 3.

In preprocessing of MR image, (i) the image is converted into greyscale, and (ii) a 3 × 3 median filter is employed on the MR image to eliminate noise that enhanced the image quality using Equation (1) [55].

(1) $$\begin{array}{c} f\left(x,y\right)={\text{median}}_{\left(s,t\right)\epsilon Sxy}\left\{g\left(s,t\right)\right\}. \end{array}$$

The obtained MR image is passed through a high pass filter to identify edges. Equation (2) provides the high-pass filter mask. After that, the edge-identified MR image is added to the original image to achieve the enhanced MR image.

(2) $$\begin{array}{c} \left[\begin{array}{ccc} -1 & 2 & -1\\ 0 & 0 & 0\\ 1 & -2 & 1 \end{array}\right]. \end{array}$$

### 3.2. Segmentation through Proposed Model

After the preprocessing step, the skull stripping is utilized to remove the skull. The purpose of skull stripping step is to separate the brain tissues from nonbrain intracranial tissues. For segmentation of tumor, the convolution layer is employed to extract the features from the MR image. The three times convolution layer and batch normalization, two times max-pooling, and four times rectified linear unit (ReLu) as an activation layer are applied in the proposed method of brain tumor segmentation. The first convolution layer with the size of the filter (kernel) 64 × 3 × 3, stride [1 1], and padding [1 1 1 1] is added to extract the features of MR images. After the convolution layer, batch normalization is applied to minimize the weight power of the nodes with high bias, to provide regularization, to improve learning speed, to normalize pixel values, to avoid overfitting, and to make the model faster. The objective of batch normalization is to align and warp image data into a general anatomical pattern before the activation function is applied. MR images for each patient from the datasets are normalized as inputs for training and testing adhering to Gaussian distribution and a variance of 1 and a mean value of 0 [46]. In Equation (3), MR images of every patient are represented by *X*. The total MR images are represented by $\hat{X}$. The mean intensity and variance of a *X* are represented by *μ* and *σ*, respectively.

Normalization is expressed as:

(3) $$\begin{array}{c} \hat{X}=\frac{X-\mu }{\sigma }. \end{array}$$

**Figure 4:**
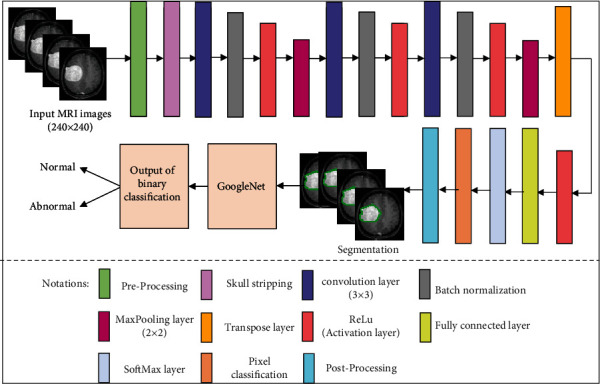
Workflow diagram of proposed framework for brain tumor segmentation and classification.

**Figure 5:**
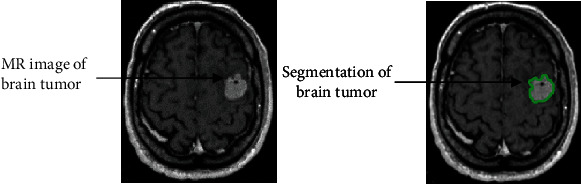
Segmentation of brain tumor using proposed methodology.

The max-pooling layer is supplied for downsampling in CNN layers and reduces feature maps at each level. The 2 × 2 max-pooling layer is selected with padding [0 0 0 0] and stride [1 1]. In other words, it decreases network's ability to identify tiny information. After that, the transpose layer is applied for the upsampling and contains many learning parameters to help create a resultant image. This layer classifies the pixels and activates the (ReLu) function. The fully connected layer is taken as the previous layer output and flattens to convert the three-dimensional matrix into the one-dimensional matrix that the next stage input. Later, Softmax transforms the input values, and the pixel classification layer is used to analyze individual image pixels by spectral information and ignores the undefined pixel labels. The workflow diagram of proposed framework is illustrated in Figure 4, and the segmented MR image of brain tumor through proposed method is shown in Figure 5.

#### 3.2.1. Postprocessing

After segmentation of MR images, the postprocessing step is applied to enhance the structural segmentation outcomes. After numerous experiments, a global threshold technique is selected to eliminate small nontumor regions based on connected components. The postprocessed segmentation outcomes are achieved by eliminating small regions and improving labels of certain pixels using a global threshold technique. Later, CNN architecture (GoogleNet) is used for the classification of brain tumor.

### 3.3. Analysis of Proposed Framework for Brain Tumor Classification

**Figure 6:**
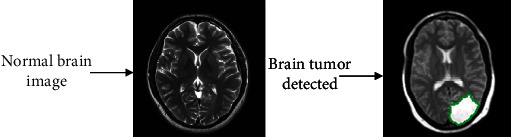
Binary classification through proposed methodology.

After segmentation, we used the CNN architecture GoogleNet to classify MR images. In this phase, qualitative analysis is performed on BRATS2018, BRATS2019, and BRATS2020 datasets for classification using the GoogleNet CNN model. The pretrained GoogleNet is used to implement transfer learning techniques like freeze layer, and fine-tune layer has significant advances for their enhanced performance on brain tumor classification. In the classification input, normal brain MR images, tumor images, and outcome consist of a binary classification shown in Figure 6.

#### 3.3.1. GoogleNet

In 2014, Bianco et al. [56] developed the CNN model (GoogleNet). He was the first ILSVRC 2014 winner who used ILSVRC datasets in his training. GoogleNet model handles the challenges of computer vision like classification to detect objects effectively. The pretrained GoogleNet architecture consists of different layers like inception module, convolutional layer, max-pooling, fully connected layer, activation function, Softmax layer, normalization layer, and some other layers. One max-pooling layer and six convolution layers are utilized for each inception module to decrease dimensions. The dropout regularization is used in a fully connected layer and ReLu activation function.

## 4. Experiments, Results, and Comparative Analysis

This section presents the proposed model, results, experiments, and comparative analysis. Our proposed brain tumor segmentation and classification model is evaluated on three (BRATS2018, BRATS2019, and BRATS2020) datasets that have actual patient data of brain tumor and attained better performance. Every patient has four modalities of the MR images (T1, T2, T1CE, and FLAIR). Our proposed model also compared with existing segmentation and classification techniques, including AFPNet [46], CRFs [41], VGG-16 [57], 3D-Dense UNets [58], GLCM [59], and *T*-test technique [45] which are evaluated on brain tumor segmentation and classification on BRATS dataset.

### 4.1. BRATS Datasets

In this work, three MACCAI benchmark challenges on multimodal brain tumor datasets BRATS2018 [60], BRATS2019 [61], and BRATS2020 [21] are used in the proposed framework. The dimension of MR images in BRATS datasets is 240 × 240 pixels. These three datasets [21] are used for segmentation and binary classification into three segments for training, validation, and testing. In the proposed model, 70% of the training data is used to learn the model. The 10% validation data is utilized for model evaluation, and model parameters tuning the 20% data are used for testing. Four modalities (T1, T2, T1CE, and FLAIR) are scanned in the dataset for each patient. BRATS2018 dataset contains total of 1425 MR images, in which 998 MR images for training, 142 MR images for validation, 285 MR images for testing are used and included total MR images of four modalities 356 T1, 355 T2, 356 T1CE, and 358 FLAIR. The BRATS2019 consists of a total of 1675 MR images, in which 1173 MR images are used for training, 167 MR images for validation, and 335 MR images for testing used and included total MR images of modalities 418 T1, 419 T2, 419 T1CE, and 419 FLAIR. The BRATS2020 dataset consists of total MR images 2470, in which 1729 MR images are used for training, 247 MR images for the validation, and 494 MR images for testing phase and included total MR images of modalities 616 T1, 616 T2, 617 T1CE, and 618 FLAIR. Various parameters such as accuracy, sensitivity, specificity, precision, and dice score are applied to test MR images dataset. All characteristics of three BRATS datasets are shown in Table 1.

### 4.2. Training Details

In training, we have used cross-validation techniques for measuring the performance of the training period. Two different methods are used to train the data that contains 10-fold cross-validation. The first technique divided the data into ten equivalent regions, so the tumor is equally available in each section, represented as recorded cross-validation [57]. Another method was utilized to arbitrarily split the data into ten equivalent sections in which data could only be found from one subject. Thus, each package included data from many subjects irrespective of the brain tumor class identified as subject-wise cross-validation. This technique is applied to assess the network capacity to generalize medical diagnoses. The capacity for generalization in clinical practice means that the diagnosis can be predicted based on evidence collected from subjects on which there are no findings during training. The focal loss function in Equation (4) is applied to resolve class imbalance problems. The focal loss is provided as weights to pixels, in which *k* signifies the number of classes, which indicates that the pixels belong to the *k*th class, and *P*_*k*_ is the predicted probability, and *p* indicates a high probability that is easier to classify accurately [62]. The focal loss function value is 10, and weights are allocated based on the complexity that classifies the pixels effectively.

(4) $$\begin{array}{c} L_{\text{Focal}}=-{\left(1-P\right)}^{\gamma }\overset{k}{\underset{k-1}{\sum }}l_{k}*\ln\left(P_{k}\right). \end{array}$$

We divided our MR image data into training, validation, and testing. The proposed framework is trained by a minibatch size 30, Adam optimizer during training, learning rate 0.001, and the data is shuffled in each iteration. This study employs a Glorot initializer, also called Xavier initializer, for the weights of the convolutional layers. Five performance matrices (accuracy, specificity, recall, precision, and dice score) are used to evaluate the performance. These performance matrices took the training time of 33 minutes and 19 sec with the proposed model. Our proposed model is taken as 99.95 sec, the average training time per epoch. The experimental parametric selection is shown in Table 2.

**Figure 7:**
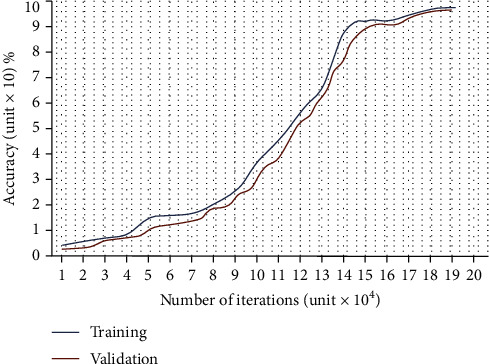
Training and validation accuracy (*y*-axis) of GoogleNet regarding the number of iterations (*x*-axis).

**Figure 8:**
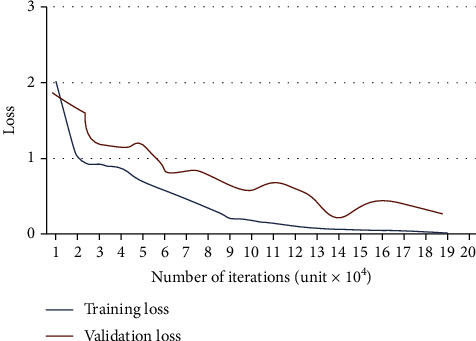
Loss curves.

The graph of training and validation accuracy (*y*-axis) of GoogleNet regarding the number of iterations (*x*-axis) is illustrated in Figure 7, and the loss curve is shown in Figure 8. We found that the network rapidly started learning from MR images in all iterations from curves. When 25% of the training data was utilized, we found that the training loss decreased, although the validation loss increased. The training cycle ends until the validation losses are higher than the previous negligible loss ten times. In the last iteration, GoogleNet achieves the maximum accuracy, later flattening the curve.

### 4.3. Model Implementation

In this work, we have implemented our proposed model in Python language. Python's TensorFlow (open-source high-level) DL library is used to implement the model [63]. The experiment has been performed on Windows 64-bit CPU with Core i9, 10^th^ generation, and NVIDIA RTX 3090 GPU with 24 GB RAM to train and validate the data.

### 4.4. Confusion Matrix

**Figure 9:**
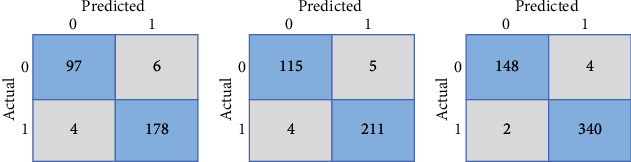
Confusion matrix of (a) BRATS2018 dataset, (b) BRATS2019 dataset, and (c) BRATS2020 dataset for classification performance of the GoogleNet model.

A confusion matrix has represented the predictions of the framework, in which each row represents the actual class, and the predicted class represents each column. A more profound visual representation of the class is misclassified, and the outcome of values divided by the number of entries in every category provides a standard confusion matrix. According to this confusion matrix, 275 MR images, 326 MR images, and 488 MR images of brain tumor are classified accurately into BRATS2018, BRATS2019, and BRATS2020 datasets. The confusion matrices of BRATS datasets for the GoogleNet classifier are shown in Figure 9.

### 4.5. Performance Metrics

We have computed our model with validation results and five evaluation parameters. True positive (TP) and true negative (TN) values are classified as correct, where TP indicates accurately classified abnormal brain images and TN indicates accurately classified normal brain images. In contrast, false positive (FP) and false negative (FN) are classified as incorrect, and FP shows incorrect typical brain images, and FN means incorrect abnormal brain images [64]. We have evaluated our proposed model on the accuracy, recall/sensitivity, specificity, precision, and dice score/*F*1-score using the following equations.

(5) $$\begin{array}{c} \text{Accuracy}=\frac{\text{TP}+\text{TN}}{\left(\text{TP}+\text{FN}+\text{TN}+\text{FP}\right)}\times 100 \end{array}$$

(6) $$\begin{array}{c} \text{Sensitivity or Recall}=\frac{\text{TP}}{\text{TP}+\text{FN}}\; \end{array}$$

(7) $$\begin{array}{c} \text{Specificity}=\frac{\text{TN}}{\text{TN}+\text{FP}} \end{array}$$

(8) $$\begin{array}{c} \text{Precision}=\frac{\text{TP}}{\text{TP}+\text{FP}} \end{array}$$

(9) $$\begin{array}{c} \text{Dice score or }F1-\text{score}=2\times \frac{\text{Precision}\times \text{Recall}}{\text{Precision}+\text{Recall}} \end{array}$$

**Figure 10:**
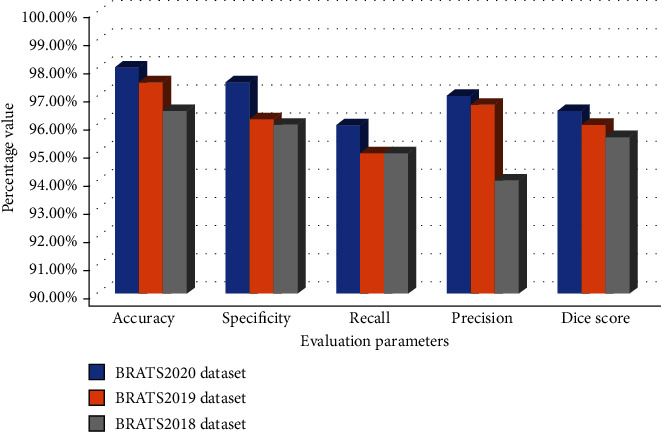
Graphical representation of performance measures for brain tumor segmentation.

**Figure 11:**
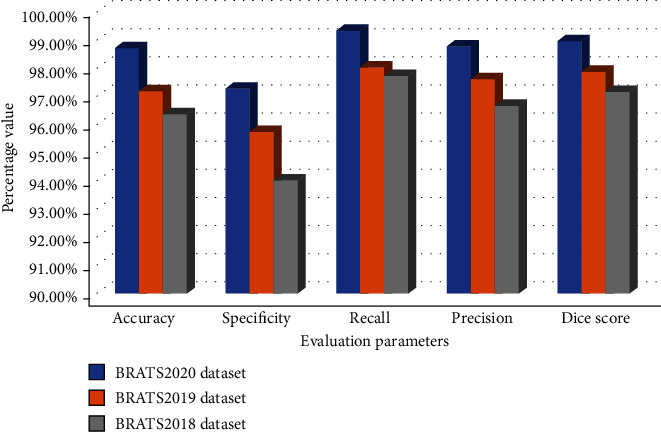
Graphical representation of performance measures for brain tumor classification.

The proposed method results by using the in-depth CNN features are obtained with high performance. This is an indicator that samples with a brain tumor are appropriately classified. The evaluation parameters are taken with the proposed model's training time of 33 minutes and 19 sec. The graphical representation of the evaluation parameters and comparison of the three datasets with the GoogleNet classifier for brain tumor segmentation and classification are shown in Figures 10 and 11.

### 4.6. Results

The proposed framework outcomes for brain tumor classification and segmentation are described in this section. The proposed method is achieved maximum batch accuracies of 96.50%, 97.92%, and 98.79%, and minimum batch accuracies of 95%, 96.50%, and 98% on BRATS2018, BRATS2019, and BRATS2020 datasets, respectively. The proposed method shows average accuracies of 96.50% for images of BRATS2018 dataset, 97.50% for images of BRATS2019 dataset, and 98.00% for images of BRATS2020 dataset for brain tumor segmentation. Similarly, the proposed method shows average accuracies of 96.49% for images of BRATS2018 dataset, 97.31% for images of BRATS2019 dataset, and 98.79% for images of BRATS2020 dataset for brain tumor classification. The results show that the highest accuracy is achieved on the BRATS2020 dataset for brain tumor classification. The error rate and the computational time are attained 3.02% on the BRATS2020 dataset. The detailed results of proposed method with standard deviation for brain tumor segmentation are shown in Table 3, and the detailed results with standard deviation for brain tumor classification are presented in Table 4.

### 4.7. Comparison with State-of-the-Art Methods

The performance of the proposed framework has been compared with some previous methods. The proposed method is compared with its baseline of FCNN, CRF, and other networks included 3D-Dense-UNets and PSO algorithm. Table 4 provides an extensive comparison performed on BRATS2018 dataset. The outcomes exhibit that the developed framework offers significantly better performance as compared to the other research studies [41, 42, 46, 47, 50]. The state-of-art relative analysis of the model implies that the developed model is dominant and surpasses. The comparative analysis of the proposed model with existing models for brain tumor segmentation is shown in Table 5.

## 5. Discussion

This paper presents a DL method based on FCNN and CRFs for the segmentation of brain tumor. The transfer learning techniques are employed on GoogleNet model to classify the MR images, and preprocessing and postprocessing are performed for better results of proposed model. The proposed framework contains two main stages: segmentation and classification, which provide an efficient and reliable method for brain tumor detection. The sophisticated and accurate outcomes required a large amount of data to train the model. Therefore, three diverse datasets (BRATS2018, BRATS2019, and BRATS2020) are utilized to train and test the proposed model that consists of binary classification. The CNN architecture GoogleNet has built on interrelated modules, which developed with our proposed model. The proposed model appears to work well on low-contrast tumor regions, as evidenced by the analysis.

The SbDL model was proposed by Sharif et al. [50] for brain tumor segmentation. This method could not achieve higher accuracy due to weak feature extraction. Leksell Gamma Knife device for the treatment of brain lesions and fuzzy C-means approach used for brain lesion segmentation presented by Militello et al. [65] showed good results with 95.59% similarity index, 97.39% sensitivity, and 94.30% specificity. The segmentation was performed on 15 patients' MR image datasets. Their proposed method is useful for supporting the planning phases of neuroradiosurgery treatment. Rundo et al. [66] developed a method based on fuzzy C-means algorithm for identification and extraction of necrosis (NeXt). Their dataset consisted of 32 brain metastatic tumors in which presented 20 tumors necrosis. The outcomes showed that DSC was 95.93% on 10 patient's datasets. However, this method was tested on a smaller dataset. The testing dataset should be enhanced to validate the performance of the model. Hence, our proposed framework caters better results than above-discussed methods.

The advantages of our developed method are (i) the segmentation outcomes on five metrics (accuracy, recall, dice score, specificity, and precision) are comparable to the radiologist; (ii) the proposed framework not only segments the entire brain tumor with low contrast MRI scans but is also computationally efficient and can potentially save lives; (iii) the proposed method is based on the DL approach and a fully automated system without user involvement. The related work section discusses the various segmentation and classification strategies of brain tumor using MRI. To validate the effectiveness and robustness of our method, we also compared the results obtained by the developed model with state-of-the-art methods on three BRATS datasets. The results demonstrate that the performance of our developed framework improves with segmentation and classification than all state-of-the-art methods.

## 6. Conclusion

The DL-based model is proposed for automated segmentation and classification of brain tumor. The brain tumor is efficiently and accurately detected through MR images using the proposed framework. Preprocessing and postprocessing steps are used to improve low contrast MR images using segmentation. Moreover, deep transfer learning techniques are used to extract features from brain MR images to enhance performance. A CNN architecture, GoogleNet, has been used for the classification of MR images. In the proposed model, three datasets (BRATS2018, BRATS2019, and BRATS2020) are utilized to train and validate brain tumor detection with highest efficacy. The experimental results of the proposed methodology showed on these three datasets have attained the maximum batch accuracies of 96.50%, 97.92%, and 98.79%, and minimum batch accuracy of 95%, 96.50%, and 98%, respectively. In the proposed methodology, the accuracies have been achieved on BRATS2018, BRATS2019, and BRATS2020 datasets, 96.50, 97.50%, and 98% for the brain tumor segmentation, and 96.49%, 97.31%, and 98.79% for the brain tumor classification, respectively. Therefore, our model takes less computational and execution time. The error rate and the computational time have attained 3.02% on the BRATS2020 dataset.

Furthermore, we have also compared the proposed methodology with some existing models. The findings indicate that the proposed framework has improved performance and is significantly better than the previous methods. The comparison of the results with current work in the literature provides evidence of the novelty and efficiency of the developed methodology. We conclude that our proposed method has achieved better accuracy with a low error rate from the results. Our proposed model performs a predictive significance in the detection of tumors in brain tumor patients. The proposed model is employed for segmentation that segments the tumor area and then performs classification. The proposed framework will be utilized in the medical field and help doctors and clinicians related to brain tumor diseases.

In future work, the proposed framework can be extended for multiclassification of brain tumor such as pituitary, glioma, and meningioma and perhaps may be useful to detect other brain abnormalities. Many possible directions to address these challenges could be considered, such as deep supervision.

## Figures and Tables

**Table 1:** MR images detail in BRATS datasets.

Dataset name	Dataset size	Brain tumor types	Data partitioning	MR images modalities			
				T1	T2	T1CE	FLAIR
BRATS2018	Total of 1425 MR images	1050 HGG, 375 LGG	998 training MR images	250	249	249	250
			142 validation MR images	35	35	36	36
			285 testing MR images	71	71	71	72
BRATS2019	Total of 1675 MR images	1295 HGG, 380 LGG	1173 training MR images	293	293	293	294
			167 validation MR images	41	42	42	42
			335 testing MR images	84	84	84	83
BRATS2020	Total of 2470 MR images	1435 HGG, 645 LGG, 390 unknown grades	1729 training MR images	432	432	432	433
			247 validation MR images	61	62	62	62
			494 testing MR images	123	123	124	124

**Table 2:** Experimental parametric selection.

Proposed model	Parameters selection	Values
CNN-based model for segmentation	Initial learning rate	0.001
	Minimum batch size	30
	Learning algorithm	Adam optimizer
	Focal loss function	10
	Maximum epochs	20
	Iterations	10,000

**Table 3:** Proposed method results with standard deviation for brain tumor segmentation.

Datasets	Accuracy	Specificity	Recall	Precision	Dice score
BRATS2018	96.50 ± 0.15	96.00 ± 0.25	95.00 ± 0.07	94.00 ± 0.02	95.50 ± 0.13
BRATS2019	97.50 ± 0.09	96.20 ± 0.15	95.00 ± 0.16	96.70 ± 0.16	96.00 ± 0.22
BRATS2020	98.00 ± 0.15	97.50 ± 0.18	96.00 ± 0.25	97.00 ± 0.05	96.50 ± 0.04

**Table 4:** Proposed method results with standard deviation for brain tumor classification.

Datasets	Accuracy	Specificity	Recall	Precision	Dice score
BRATS2018	96.49 ± 0.08	94.17 ± 0.07	97.80 ± 0.21	96.74 ± 0.09	97.27 ± 0.19
BRATS2019	97.31 ± 0.17	95.83 ± 0.18	98.14 ± 0.09	97.69 ± 0.12	97.92 ± 0.27
BRATS2020	98.79 ± 0.23	97.37 ± 0.25	99.42 ± 0.02	98.84 ± 0.16	99.12 ± 0.15

**Table 5:** Comparative analysis of proposed framework with state-of-art methods for brain tumor segmentation.

Ref no.	Author	Year	Technique	Dataset	Results
[41]	Hu et al.	2019	MCCANN, CRFs	BRATS2018 dataset	Dice score for ET, WT, and TC was 71.78, 88.24, and 74.81; sensitivity for 86.84, 90.74, and 76.21; specificity for 99.47, 99.18, and 99.69, respectively
[42]	Zhou et al.	2020	AFPNet, 3D CRF	BRATS2018 dataset	Lesion structure for ET 74.43, WT 86.58, and TC 76.88
[46]	Akil et al.	2020	Based on CNN	BRATS2018 dataset	MDS for WT 90.00, CT 83.00, and ET 83.00
[47]	Bangalore et al.	2020	3D-dense-UNets	BRATS2018 dataset	Dice score for WT 90.00, TC 82.00, and ET 80.00
[50]	Sharif et al.	2020	DRLBP, PSO algorithm	BRATS2018 dataset	Dice score for CT 88.30, for WT 91.20, for ET 81.80, and accuracy > 92.00
Proposed method for segmentation		2021	Based on FCNN and CRFs	BRATS2018 dataset	Dice score 95.50 ± 0.13, accuracy 96.50 ± 0.15
				BRATS2019 dataset	Dice score 96.00 ± 0.22, accuracy 97.50 ± 0.09
				BRATS2020 dataset	Dice score 96.50 ± 0.04, accuracy 98.00 ± 0.15

## Data Availability

The brain MR image data used to support the findings of this study is publicly available in the repositories ([https://www.kaggle.com/anassbenfares/brats2019-1], [https://www.kaggle.com/vahidehghobadi/brats2018], [https://www.kaggle.com/awsaf49/brats20-dataset-training-validation]).
